# Vitrectomy with amniotic membrane transplantation for macular choroidal coloboma with macular hole and posterior scleral staphyloma: a case report

**DOI:** 10.3389/fmed.2025.1731661

**Published:** 2026-01-02

**Authors:** Jie Liang, Shanjun Cai

**Affiliations:** 1 Department of Ophthalmology, Affiliated Hospital of Zunyi Medical University, Zunyi, China; 2 Zunyi Medical University, Zunyi, China

**Keywords:** amniotic membrane transplantation, macular choroidal coloboma, macular hole, macular posterior scleral staphyloma, vitrectomy

## Abstract

**Background:**

This study aimed to report a case of vitrectomy with amniotic membrane transplantation (AMT) for a macular choroidal coloboma (CC) associated with a macular hole (MH) and posterior scleral staphyloma (PSS).

**Case presentation:**

A 54-year-old woman presented with a 10-day history of decreased vision and metamorphopsia in her right eye. Other than bilateral cataracts, no other ocular abnormalities were found. The best-corrected visual acuity (BCVA) was 0.05 in the right eye and 0.5 in the left eye. Scanning laser ophthalmoscopy (SLO) revealed an atrophic-like lesion and an MH in the macular area. Optical coherence tomography (OCT) revealed a macular CC with a full-thickness MH and PSS. Due to the large size of the MH(1238 × 976 μm) and significant ocular symptoms, the patient underwent vitrectomy with internal limiting membrane (ILM) peeling, AMT, and silicone oil tamponade (SOT). At the 3-month follow-up, the amniotic membrane (AM) was functioning, but the condition had not yet stabilized. During the subsequent silicone oil removal (SOR) procedure, a second AMT was performed, followed by an intravitreal injection of C3F8 gas. At 9 months after SOR, OCT demonstrated firm adhesion between the AM, the edges of the MH, and the sclera. The MH showed signs of stability, and the cavity formed by the CC and PSS had decreased in size. In parallel with the anatomical improvement, the patient’s visual quality improved.

**Conclusion:**

We report a rare surgical case of a macular CC with an MH and PSS. Surgery resulted in improvements in both anatomic architecture and visual quality. This case expands our understanding of the pathogenesis and management of this uncommon disease.

## Introduction

Choroidal coloboma (CC) is a congenital defect that result from incomplete closure of the embryonic fissure, with an incidence of 0.14% in the general population (1). Sporadic cases are often caused by intrauterine injury related to environmental factors, whereas hereditary cases may follow autosomal recessive, autosomal dominant, X-linked recessive, or X-linked dominant inheritance patterns (2). The colobomatous area may directly involve the optic disc and macula and can lead to complications such as retinal detachment (RD) and choroidal neovascularization (CNV), resulting in varying degrees of visual impairment. Current treatment strategies include prophylactic laser photocoagulation (PLP) along the edges of the coloboma and surgical interventions such as vitrectomy for breaks or RD (2).

Macular hole (MH) is an anatomical defect of the fovea that may lead to visual loss, central scotoma, and metamorphopsia (3). Depending on their pathogenesis, MHs can be classified as idiopathic or secondary. Secondary MHs may result from trauma, high myopia, uveitis, macular telangiectasia, macular schisis, idiopathic retinal vascular proliferative tumors, retinal vascular diseases, age-related macular degeneration, and other retinal pathologies (4). The current primary treatment is vitrectomy with or without internal limiting membrane (ILM) peeling; surgical strategies vary and should be tailored to specific anatomical conditions (3).

Posterior scleral staphyloma (PSS) is a structural abnormality characterized by outward bulging of a localized area of the posterior fundus, with a radius of curvature smaller than that of the adjacent ocular wall (5). It is considered a hallmark of pathological myopia, but it may also be observed in non-myopic patients (5). The condition may develop due to scleral thinning with localized expansion, choroidal thinning, and breaks in Bruch’s membrane (5). PSS compromises the biomechanical stability of posterior pole tissues and may predispose patients to complications such as MHs and RD (6).

We present a case of a macular CC with an MH and PSS. The patient underwent vitrectomy with amniotic membrane transplantation (AMT). Improvements in anatomic architecture and visual quality were observed following surgery.

## Case presentation

**Figure 1:**
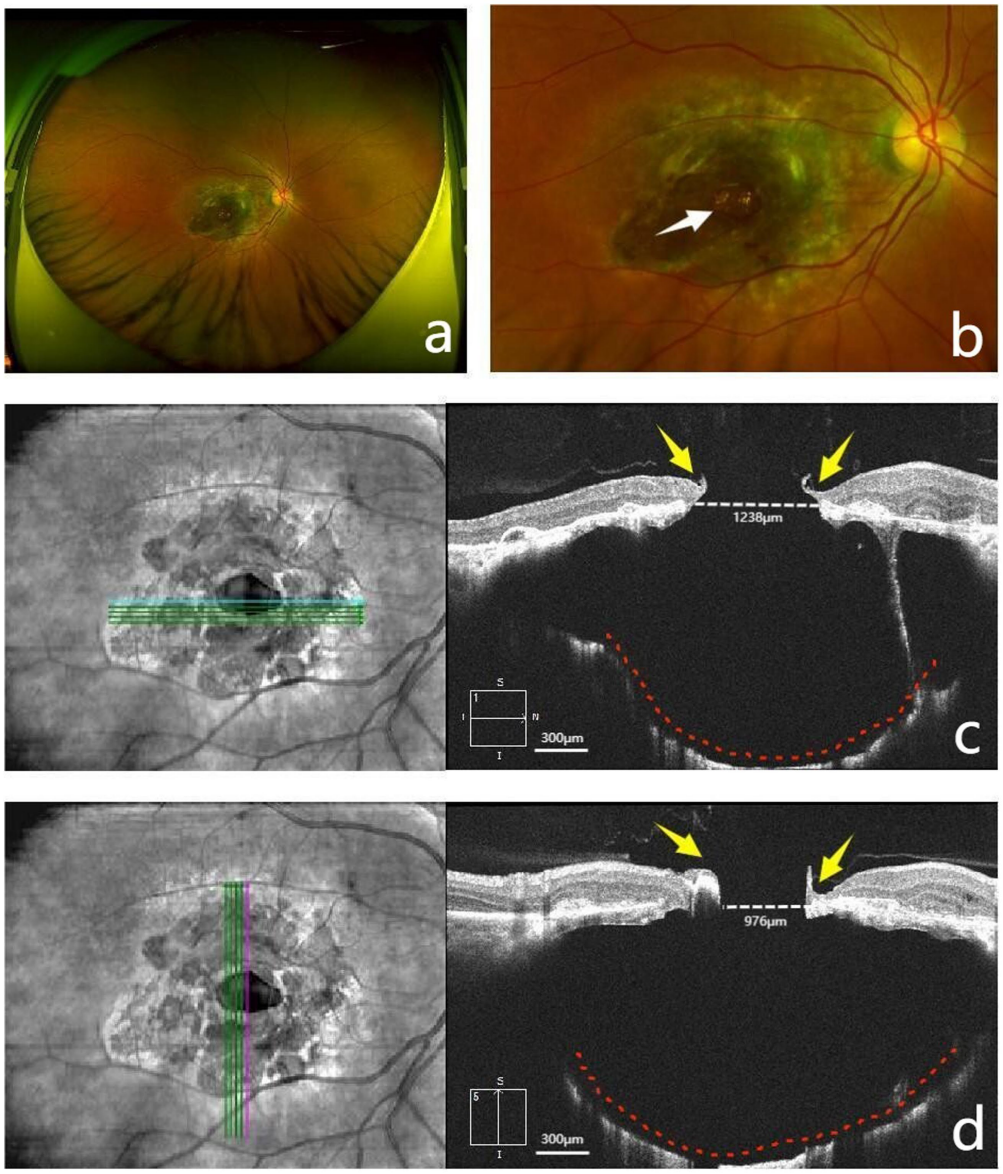
Preoperative fundus appearance. **(a)** Scanning laser ophthalmoscopy (SLO) demonstrated a well-circumscribed, roundish, yellowish-green atrophic lesion approximately 4 disc diameters in size in the macular region. **(b)** SLO revealed a well-demarcated macular hole (MH), approximately half a disc diameter in size, located in the center of the lesion (the white arrow indicates the MH). **(c,d)** Optical coherence tomography (OCT) demonstrated disorganization of the retinal layers, with an epiretinal membrane on the surface and a centrally located full-thickness MH (1238 × 976 μm). The choroid was mostly absent, with partial preservation of the choriocapillaris, while the sclera was thinned and bowed posteriorly, accompanied by posterior scleral staphyloma (PSS) (yellow arrows indicate the edges of the MH, red dashed lines outline the PSS, and white dashed lines mark the MH diameter).

A 54-year-old woman presented with a 10-day history of decreased vision and metamorphopsia in her right eye. She was initially diagnosed with an MH at another hospital and was subsequently referred to our ophthalmology department for further management. Her best-corrected visual acuity (BCVA) was 0.05 in the right eye and 0.5 in the left eye. The axial length was 21.91 mm in the right eye and 22.16 mm in the left eye. Ocular examination revealed bilateral cataracts but was otherwise unremarkable anteriorly. Scanning laser ophthalmoscopy (SLO) of the right eye showed pathological changes in the macular region (Figures 1a,b). Optical coherence tomography (OCT) revealed a macular CC with a full-thickness MH and PSS (Figures 1c,d). The final diagnosis was “macular CC with MH and PSS in the right eye”.

**Figure 2:**
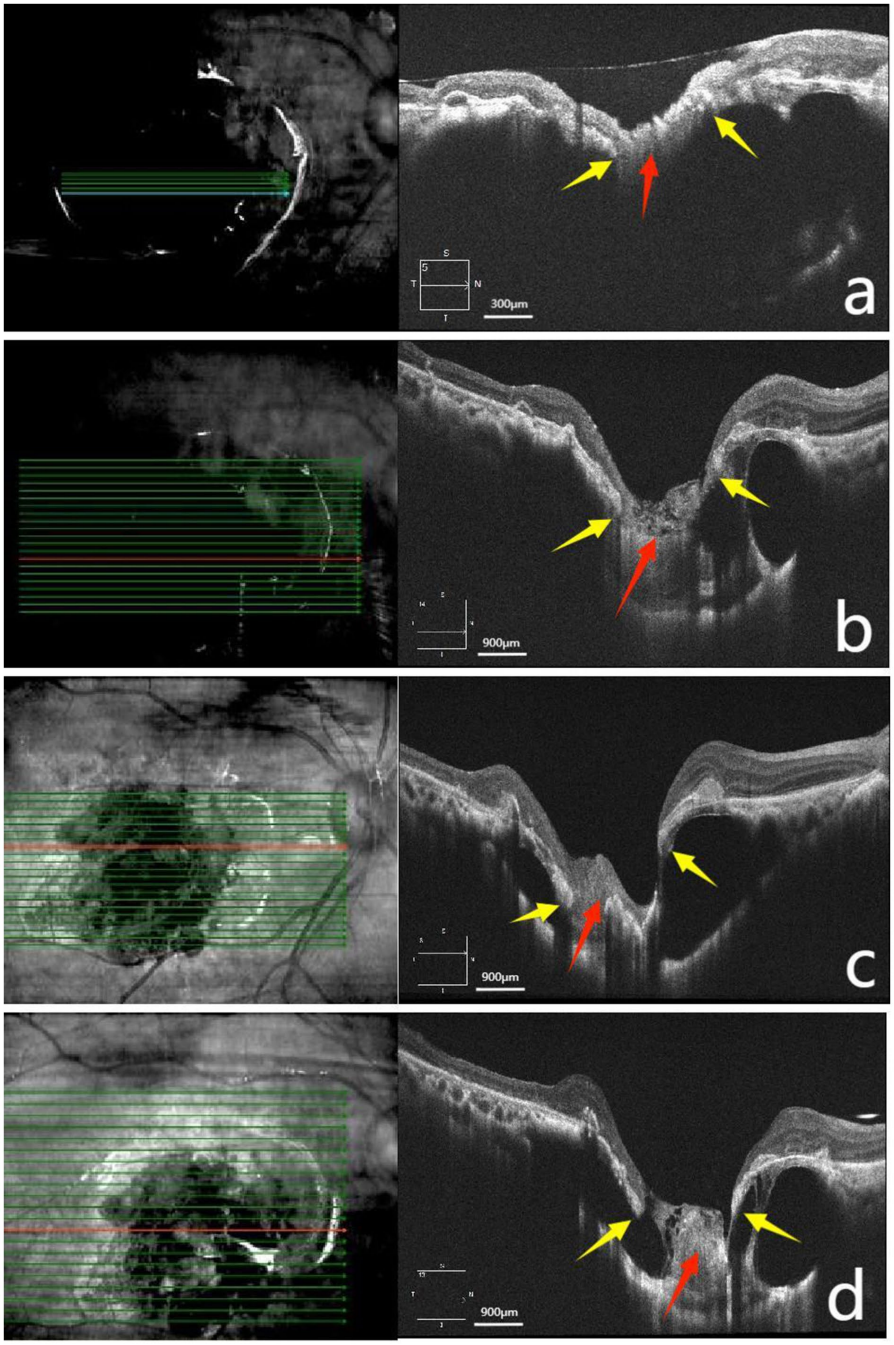
Post-vitrectomy OCT follow-up images. **(a)** Postoperative day 1: The amniotic membrane (AM) was located beneath the retinal neuroepithelium and adhered to both edges of the MH. **(b)** Postoperative day 10: The retina approximated to the sclera. The AM began to degrade, with its adhesion to the MH edges loosening. **(c,d)** Postoperative months 1 and 3: The AM continued to degrade, with its adhesion to the MH showing separation (yellow arrows in panels **a–d** indicate the edges of the MH; red arrows point to the AM).

The patient subsequently underwent surgical intervention using the Alcon Constellation Vision System (VMC212-1). Standard phacoemulsification with intraocular lens implantation was performed, followed by a three-port pars plana vitrectomy (PPV). After induction of posterior vitreous detachment, the infusion was temporarily halted, and approximately 1% indocyanine green was applied to the macular area to facilitate removal of the epiretinal membrane and a 2-disc-diameter area of the ILM. Subsequently, a thorough air–fluid exchange was performed. A cryopreserved, hydrated amniotic membrane (AM) was trimmed into an approximately 1.5 × 1.5 mm oval-shaped graft. With the relatively smooth basement membrane surface facing downward, the graft was grasped using Alcon 25G ILM forceps and positioned beneath the MH in the sub-neuroepithelial plane. Subsequently, the periphery of the MH was gently massaged using an Alcon vitreous cutter to promote close adhesion between the graft and the retinal tissue and to ensure its tissue stabilization. Finally, silicone oil was slowly and steadily injected into the vitreous cavity, taking care to avoid any direct impingement on the AM graft. The patient was instructed to maintain a face-down position for 1 month postoperatively. Postoperative follow-up with OCT showed the following progression: Postoperative day 1: The AM was *in situ* (Figure 2a); postoperative day 10: With the AM providing adhesion, the MH began to show improvement (Figure 2b); and postoperative months 1 and 3: Progressive degradation of the AM was accompanied by reduced stability of the MH (Figures 2c,d).

**Figure 3:**
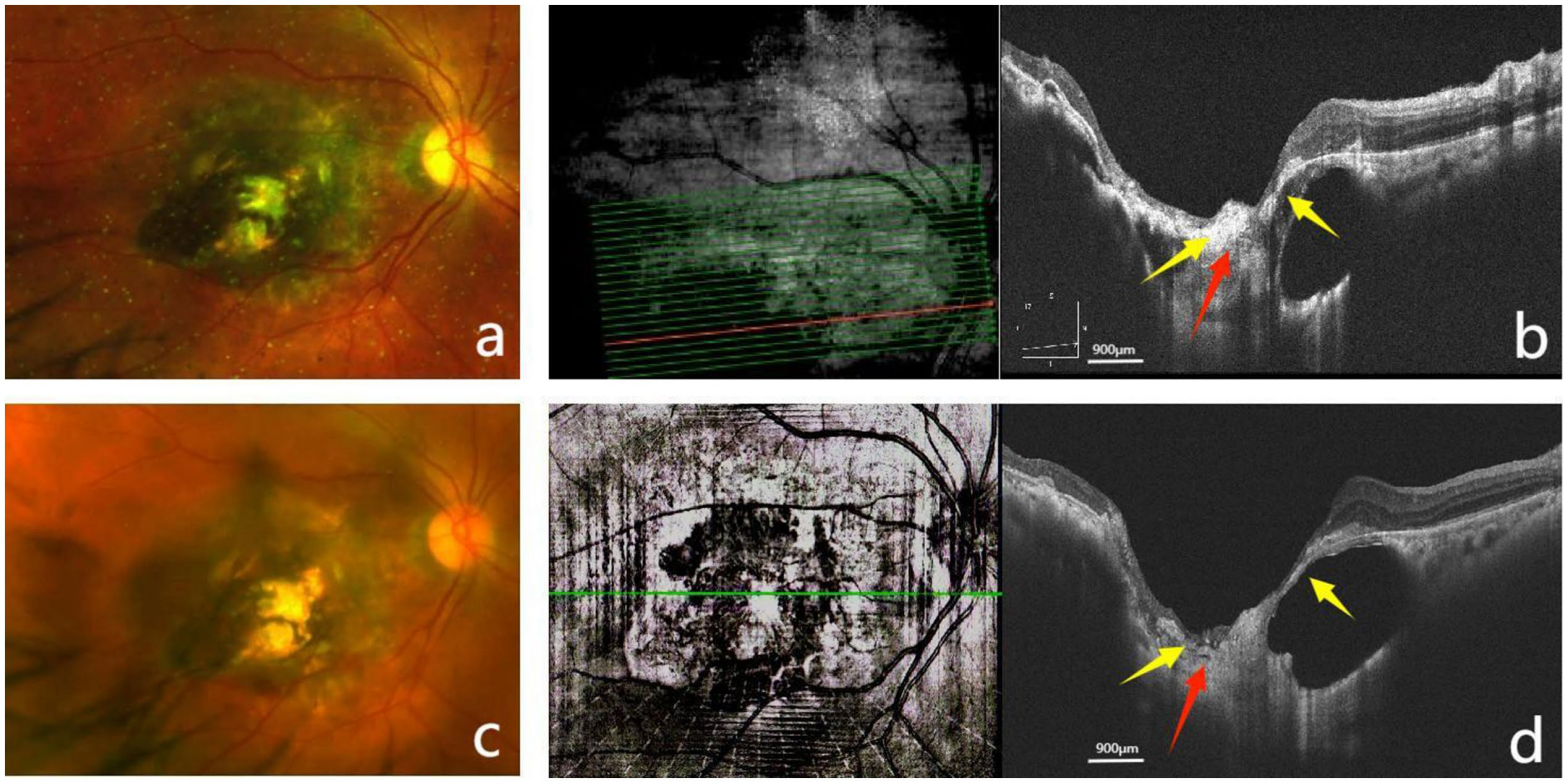
Follow-up examination after silicone oil removal (SOR). **(a)** SLO at 3 months after SOR showed the AM in position within the defect area. **(b)** OCT at 3 months after SOR showed the AM located within the region of the macular CC and PSS, firmly adherent to the sclera at its base. The edges of the MH were tightly opposed to the AM, and the cavitation associated with the CC and PSS was reduced. **(c)** SLO at 9 months after SOR showed no displacement of the AM. **(d)** OCT at 9 months after SOR showed some degree of AM degradation; however, it remained firmly adherent to both edges of the MH and the sclera. The retina remained in an approximated relationship with the sclera, showing partial apposition to the sclera (yellow arrows in panels **a–d** indicate the edges of the MH; red arrows indicate the AM).

Based on the postoperative OCT findings, the MH showed a trend toward stabilization, and the cavity formed by the CC and PSS had decreased to some extent, indicating a beneficial effect of the AM graft. However, due to membrane degradation and the extensive size of the defect, a single AMT was insufficient to fully stabilize the condition. Therefore, during the subsequent silicone oil removal (SOR) procedure, we performed a second AMT. Following the initiation of surgery, standard three-port incisions for SOR were established. An infusion cannula was inserted at the inferotemporal port, and the infusion pressure was set at 20 mmHg (1 mmHg = 0.133 kPa). The bottle spike penetrator at one end of the blood transfusion set (Model: B-0.9 × 27 TW LB; Henan Shuguang Jianshi Industrial Development Co., Ltd., Luohe City, Henan Province, China) was cut off with scissors. The truncated end was connected to the vitrectomy instrument, and the negative pressure of the vitrectomy instrument was adjusted to approximately 600 mmHg. The intravenous needle at the other end of the blood transfusion set was removed while retaining its Luer-lock connector. The plastic tube of an 18G intravenous indwelling needle (Model: IVC05-18; Ande Medical Supplies Co., Ltd., Zibo City, Shandong Province, China) was trimmed to a length of approximately 1 cm, and the cut end was beveled. This modified intravenous indwelling needle was then connected to the Luer-lock connector of the blood transfusion set. The modified plastic tube of the intravenous indwelling needle was inserted through the superotemporal sclerotomy to actively aspirate the silicone oil utilizing the negative pressure from the vitrectomy instrument. Controlled aspiration and effective flow reduction were employed to ensure tissue stabilization during the oil removal process. After complete oil removal, a thorough air–fluid exchange was performed. The periphery of the MH was then slightly separated using a vitreous cutter. Subsequently, a 1.0 × 1.0 mm cryopreserved, hydrated AM graft was grasped with ILM forceps and placed inside the MH, achieving intrahole placement. Following the AM graft insertion, gentle massage around the MH was performed using the vitreous cutter to promote tissue stabilization. Finally, C3F8 gas was injected into the vitreous cavity. The patient was instructed to maintain a face-down position for 3 weeks postoperatively. At the 3-month follow-up after SOR (6 months after the initial surgery), SLO confirmed that the AM remained in position (Figure 3a). OCT revealed that following the second AMT, the MH demonstrated structural improvement under the scaffolding effect of the AM graft (Figure 3b). At this stage, the BCVA in the right eye had improved from 0.05 to 0.1. Although the visual gain was modest, the patient reported subjective improvement in visual quality. By the 9-month follow-up after SOR (12 months after the initial surgery), the BCVA remained stable at 0.1 without regression. SLO demonstrated no displacement of the AM during the long-term follow-up period (Figure 3c). OCT revealed some degree of AM degradation under prolonged surveillance; however, the MH remained stable, while the fundus architecture demonstrated gradual improvement (Figure 3d).

## Discussion

We described an uncommon case of a unilateral CC confined to the macular region, accompanied by a full-thickness MH and localized PSS. OCT demonstrated a choroidal defect with partial preservation of the choriocapillaris. The retinal pigment epithelium (RPE) and neurosensory retina remained clearly identifiable; however, a large-aperture MH and PSS were present. These findings deviate from the classic presentation of a CC, which typically occurs in the inferonasal fundus (2). In such cases, OCT characteristically reveals a full-thickness absence of the choroid, RPE, and outer retina, with the residual inner retina eventually atrophying into a glioneuronal monolayer—the intercalary membrane (ICM) (1, 7). SLO in our case revealed a yellowish-green atrophic lesion with a central punched-out defect, resembling the fundus appearance of macular coloboma (MC). Typically, MC presents as a well-demarcated, round or oval atrophic lesion in the macula, while OCT shows hypoplasia or absence of the retina, choroid, and sclera in that region (8). Therefore, the current case appears to represent a rare syndromic association of these distinct pathologies.

What could explain the co-presentation of an MH and PSS beyond the CC? A single reported case by Venkatesh et al. described a similar combination of bilateral CC, PSS, and macular hole-related retinal detachment (9). That patient had high axial myopia, as evidenced by funduscopic chorioretinal atrophy and a confirmed PSS, although the axial length was not recorded (9). In contrast, our case had an axial length of 21.91 mm and lacked any fundus findings indicative of high myopia, thereby representing a fundamentally distinct entity. Upon re-evaluation of the patient’s medical history, there was still no report of ocular trauma, intraocular surgery, inflammation, or systemic disease. The pathogenesis of the patient’s fundus changes remained unclear, and no causal or mechanistic link within the fundus structure could be established. The characterization of these features was based solely on objective documentation. The structural damage caused by the large MH, CC, and PSS resulted in significant ocular symptoms. Prompt surgical intervention was necessary to prevent further complications and potential vision loss.

Given the complexity and rarity of this case, devising a surgical strategy necessitated a multifaceted approach. The principal goals were to achieve the closure of the MH and reduce the cavitation associated with the CC and PSS. For large MHs (particularly ≥1,000 μm), AMT has shown superior closure rates and visual outcomes compared to ILM peeling, ILM flap techniques, or autologous retinal transplantation (10). Moreover, the AM offers ideal thickness, ease of manipulation and positioning, and broad availability (11). It is suitable for a wide range of patients, including those with prior extensive ILM peeling or intraocular lens implantation (11). The patient underwent vitrectomy, ILM peeling, and AMT, with the AM graft positioned in the sub-neuroepithelial plane. This was followed by silicone oil tamponade (SOT) to ensure secure adhesion of the membrane and stabilization of the fundus architecture. At the 3-month follow-up, OCT demonstrated adhesion of the AM to both edges of the MH, with the retina approximating the sclera. The MH showed progressive stabilization, and the cavitation associated with the CC and PSS was reduced. However, due to AM degradation and the inherent complexity of the structural defects, the degree of local anatomical improvement remained limited. During the SOR procedure performed 3 months after the initial vitrectomy, a second AMT was performed. A slightly smaller AM graft was placed into the MH, achieving intrahole placement, with the aim of obtaining a more favorable prognosis. By the 9-month follow-up after SOR, the patient’s BCVA remained stable at 0.1, accompanied by subjective improvement in visual quality. OCT revealed firm adhesion of the AM to the edges of the MH and the sclera, and the retina maintained an approximated relationship with the sclera. We conclude that the MH achieved structural stability and was restored under the scaffolding effect of the AM. The cavity formed by the CC and PSS also reduced in size and remained stable over the long term. Overall, anatomical restoration of the fundus was enhanced. This surgical approach reduced the risk of further complications and prevented progressive visual deterioration in this challenging case.

The long-term outcome of the AM graft within the eye and its potential to induce other complications require further investigation through larger case series and extended follow-up. Incorporating additional examinations during follow-up—such as fundus autofluorescence or optical coherence tomography angiography to monitor changes at the RPE–choroid interface, as well as objective metrics of visual quality such as microperimetry or contrast sensitivity—would provide more comprehensive means of evaluating surgical outcomes. In the future, refinement of the surgical approach, exploration of more effective adjuvant therapies, and development of additional follow-up assessment tools may further improve treatment efficacy for these complex and rare ocular conditions while generating stronger evidence. In summary, we report a surgically managed case of a macular CC with a full-thickness MH and PSS—an exceptionally rare presentation. The intervention led to both anatomical and visual quality improvements, thereby expanding our understanding of the pathogenesis and management of this uncommon disease.

## Data Availability

The original contributions presented in the study are included in the article/supplementary material, further inquiries can be directed to the corresponding author.
